# SigWinR; the SigWin-detector updated and ported to R

**DOI:** 10.1186/1756-0500-2-205

**Published:** 2009-10-06

**Authors:** Wim C de Leeuw, Han Rauwerda, Márcia A Inda, Oskar Bruning, Timo M Breit

**Affiliations:** 1 MicroArray Department & Integrative Bioinformatics Unit, Swammerdam Institute for Life Sciences, Faculty of Science, University of Amsterdam, Science Park 904, 1098 XH Amsterdam, the Netherlands

## Abstract

**Background:**

Our SigWin-detector discovers significantly enriched windows of (genomic) elements in any sequence of values (genes or other genomic elements in a DNA sequence) in a fast and reproducible way. However, since it is grid based, only (life) scientists with access to the grid can use this tool. Therefore and on request, we have developed the SigWinR package which makes the SigWin-detector available to a much wider audience. At the same time, we have introduced several improvements to its algorithm as well as its functionality, based on the feedback of SigWin-detector end users.

**Findings:**

To allow usage of the SigWin-detector on a desktop computer, we have rewritten it as a package for R: SigWinR. R is a free and widely used multi platform software environment for statistical computing and graphics. The package can be installed and used on all platforms for which R is available. The improvements involve: a visualization of the input-sequence values supporting the interpretation of Ridgeograms; a visualization allowing for an easy interpretation of enriched or depleted regions in the sequence using windows of pre-defined size; an option that allows the analysis of circular sequences, which results in rectangular Ridgeograms; an application to identify regions of co-altered gene expression (ROCAGEs) with a real-life biological use-case; adaptation of the algorithm to allow analysis of non-regularly sampled data using a constant window size in physical space without resampling the data. To achieve this, support for analysis of windows with an even number of elements was added.

**Conclusion:**

By porting the SigWin-detector as an R package, SigWinR, improving its algorithm and functionality combined with adequate performance, we have made SigWin-detector more useful as well as more easily accessible to scientists without a grid infrastructure.

## Introduction

For the detection of significantly enriched windows of elements in any sequence of values in a fast and reproducible way, we developed and published a workflow and grid-based tool; SigWin-detector[1]. For instance, elements may be genes and a sequence may consist of values attributed to these genes. SigWin-detector is based on a moving median false discovery rate (mmFDR) procedure using an exact formula. SigWin-detector visualizes significantly enriched windows by Ridgeograms; the sequence is depicted by stacking increasing window sizes from 1 onward, thus forming a triangle. Enriched or depleted windows are marked by a color. Windows in the input sequence are considered to be significantly enriched, if they have a median value that deviates significantly from the expected value assuming random ordering of the values in the input sequence. The development of SigWin-detector was originally motivated by the need to identify regions of increased gene expression (RIDGEs) in the human transcriptome map (HTM) [1,2], see Additional file 1. However, the applicability of the tool is much wider, as we discovered that SigWin-detector can also be used to identify regions of co-altered gene expression (ROCAGE).

Because SigWin-detector has been implemented on a grid-platform, and many life scientists do not have access to grid resources, we have received requests from users for a SigWin-detector that operates in a non-grid environment. We therefore have ported our SigWin-detector to R [3], the most commonly used statistical language in omics research. At the same time, we have extended the underlying algorithm and the functionality of SigWin-detector. The R package is called SigWinR.

## Description

### Porting SigWin-detector to R

The original SigWin-detector workflow was rewritten in the R language, except for the median calculation of the moving windows, which was programmed in C to achieve an acceptable performance. SigWinR can produce a Ridgeogram for a sequence containing 10,000 elements in less than 1 minute using a modern desktop computer with an Intel^® ^Core™ 2 Quad Q8200 Processor running at 2.33 GHz with 2 GB of RAM. Hence, it is feasible to analyze whole eukaryotic genomes within a practical timeframe. SigWinR has been developed on R-2.8.0 and has been tested on a Linux and Microsoft Windows environment. The package has been validated with the data sets used in [1] (results not shown). Help and documentation is available in the R package, which can be downloaded from the Comprehensive R Archive Network (CRAN, ).

### Visualizing input-sequence values

Ridgeograms are the standard output of SigWinR. To support the interpretation of the produced Ridgeogram, we have added a XY-plot below the Ridgeogram containing the values of all elements in the sequence. All Figures show examples of this visualization.

### Visualizing enriched windows of pre-defined size

**Figure 1:**
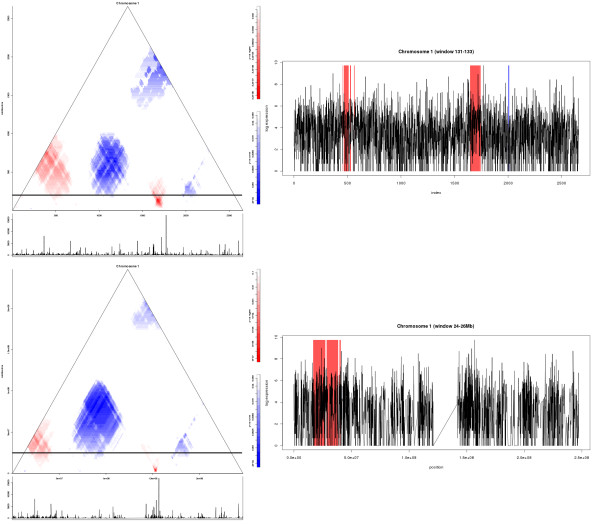
**Advanced SigWinR Ridgeogram for RIDGES in the human transcriptome map**. An example of SigWinR Ridgeograms (left) and Ridgeplots for pre-defined window sizes (right) for chromosome 1 from the analysis of (anti-)RIDGEs in the human transcriptome map (HTM) [2]. The p-values of the RIDGEs (red) and anti-RIDGES (blue) in the Ridgeograms are indicated by the shade of the colour as shown on the scale bars to the right of the Ridgeograms. Below the Ridgeograms, an XY-plot of the gene-expression values of the whole genome sequence is depicted. In the Ridgeplots the stretches with p-values (corrected for multiple testing) below 0.01 are shown: RIDGES with a red shade, anti-RIDGES with a blue shade. The window size taken to generate the Ridgeplots is indicated by a horizontal bar in the Ridgeograms. The XY-plots beneath the Ridgeogram are shown in non-log space, whereas in the Ridgeplots log values are used. Since the Sigwin algorithm is based on a rank statistics, this is of no influence to the result. Upper part: The input sequence is the genes ordered by their occurrence on the chromosomes. The value of the sequence elements is gene-expression from a serial analysis of gene expression (SAGE) compendium. Lower part: An example of a SigWinR PosRidgeogram for essentially the same analysis, but with an input sequence consisting of 1 kb chromosomal stretches. As such, although the moving window analyses for every line in the Ridgeogram the same chromosomal size, it will contain a variable number of genes. In the Ridgeplot the centromere is clearly visible as a region with a low sample density. The Ridgeograms of all chromosomes from these analyses are presented in the Additional files 1 and 4, respectively.

Ridgeograms are visualizations of significant windows using all possible window sizes. However, it often occurs that the most interesting scale on which to analyze a sequence is known. For those cases, we have added an extra option in SigWinR that allows identification of significantly enriched windows for a pre-defined subset of window sizes. This makes the analysis considerably more efficient. In Figure 1 (upper and lower right panel) an example of this visualization is shown.

### Analyzing circular sequences

**Figure 2:**
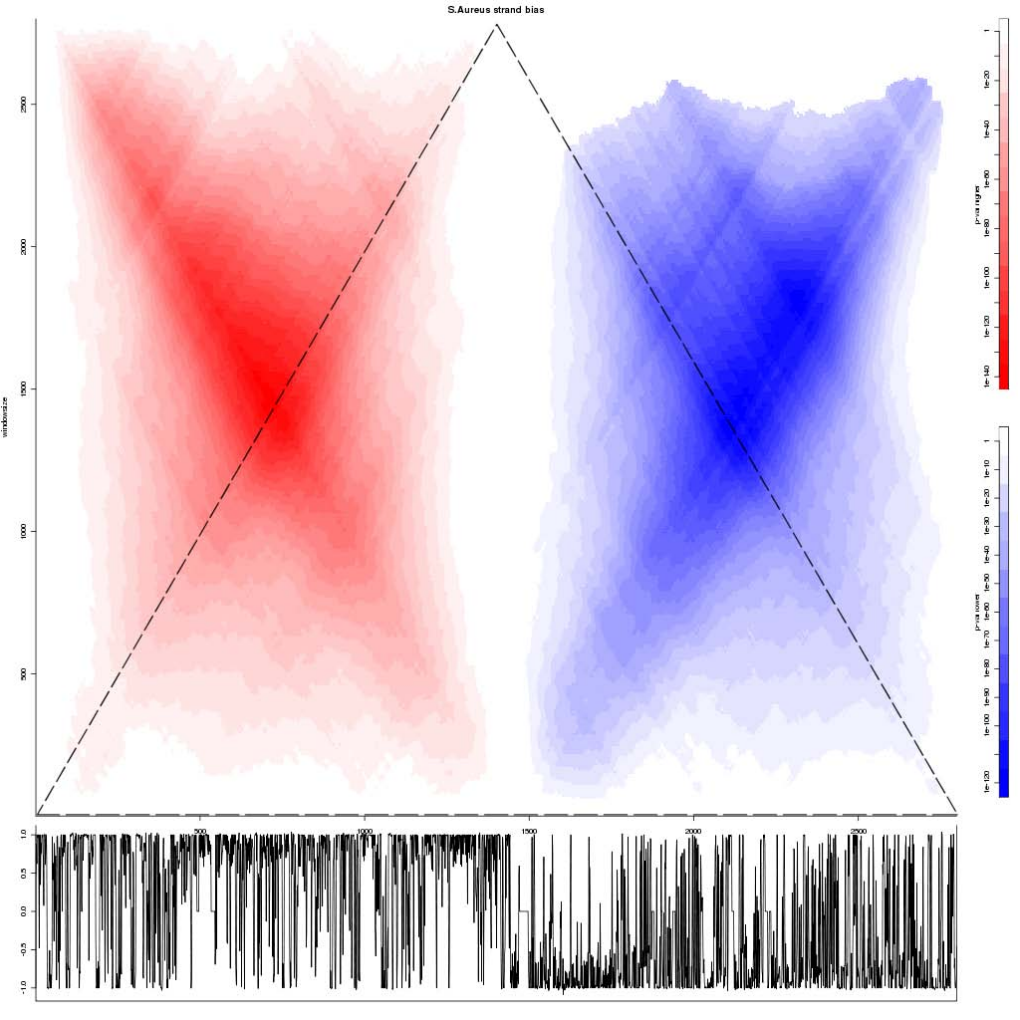
**SigWinR rectangular Ridgeogram of the gene density in the circular bacterial genome of Staphylococcus aureus**. The SigWinR rectangular Ridgeogram showing the significant windows of gene density, i.e. the number of nucleotides that belong to a gene, on the DNA positive strand of the circular genome of bacterium Staphylococcus aureus using a bin-size of 1 kb. The input values are calculated per 1 kb bin as the difference of the number of gene nucleotides between the top strand and the bottom strand divided by 1,000. The DNA origin-of-replication (ori) and replication terminus (ter) are at position 0 and ~1,500 at the X-axis, respectively. The Y-axis shows the window size. The known, skewed gene distribution with over-representation in the DNA replication leading strand (ori → ter) and under-representation in the lagging strand (ter → ori) [5,6], are clearly visible and most pronounced at a window size of half the total sequence length. Furthermore, the Ridgeogram shows that on the leading strand near the ter, the gene density is highest. The dotted triangle is placed in this figure to illustrate which part of this rectangular Ridgeogram represents the content of the common (triangular) Ridgeogram. Below the Ridgeogram, a XY-plot of the 1 kb bin gene-density values of the whole genome sequence is depicted.

Since SigWin-detector originates from the life sciences, an obvious extension of SigWinR is the possibility to analyze circular sequences, such as bacterial genomes. As in a linear sequence, the largest meaningful size of the moving window in a circular sequence equals the total length of that sequence. However, where in linear sequences the moving windows decrease in number as they increase in size (hence the triangular shape of the Ridgeogram), in a circular sequence, all possible window sizes including the largest, can still travel across the entire sequence. Therefore the Ridgeogram is rectangular. An example of an analysis of a circular sequence is shown in Figure 2. Relevant observations could be missed, if circular nucleotide sequences are analyzed as artificially linearized sequences based on an arbitrary cut, such as the origin of replication in bacterial genomes.

### Identifying elements with altered values that are co-localized in a sequence

**Figure 3:**
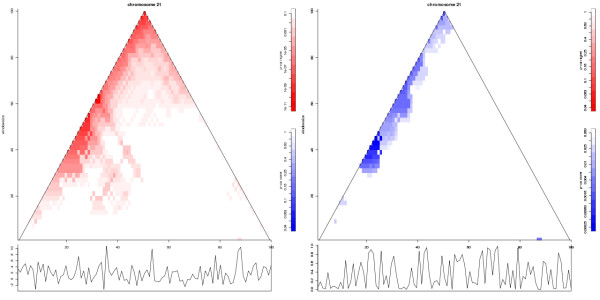
**SigWinR Ridgeogram identifying ROCAGES in Down syndrome data**. Ridgeograms of human chromosome 21 from the SigWinR analysis of Down syndrome (trisomy 21) transcriptome data [4]. Left: Input-sequence values are the per gene t-values, i.e fold change of gene-expression log ratios of Down and control samples divided by their standard error. Right: Input-sequence values are the p-values, which indicate the statistical significance of the gene-expression differences between Down and control samples. The data for all chromosomes concatenated has been used as input for the analysis, the result for chromosome 21 was extracted from the genome wide Ridgeogram. Using both types of input-sequence value results in detection of regions of co-altered gene expression (ROCAGEs). As expected, both Ridgeograms show clear ROCAGEs in the analysis of the chromosomal trisomy typifying this syndrome. The Ridgeograms of all chromosomes from these analyses are presented in the Additional files 2 and 3, respectively, the R code and data used to generate the figures can be downloaded from the project home page.

In life sciences, it is often interesting to identify regions in a sequence in which many of the elements have an altered value (ROCAGEs) in the context of an experimental contrast. Thus, when using for instance gene-expression data, instead of identifying RIDGES, which requires data from many experiments, we would like to be able to identify ROCAGEs within single experiments. To accomplish this, SigWin-detector can be fed an input sequence consisting of gene-expression log ratios or, in a replicated experiment per gene t-values or per gene p-values. As an example we investigated gene-expression data concerning Down syndrome that is typified by a trisomy of chromosome 21 (Figure 3 and Additional files 2 and 3) [4]. In chromosomal regions that are duplicated such as the Down syndrome chromosome 21, one expects to find co-localized genes with altered gene expression. Indeed Figure 3 and Additional files 2 and 3 show ROCAGES for the Down chromosome 21 vs. control tissue using t-values and p-values as input.

### Considering the spatial distribution of sequence elements

The spatial distribution of genes on chromosomes is not uniform. In the previous implementation [1] this was solved by re-sampling the input sequence, which distorts the data. Here we have implemented a method in which the distribution is taken into account by representing the data as a non-regularly sampled sequence. This sequence is a sequence of position, value pairs. In SigWinR, a new function (PosRidgeogram) is available, which calculates a Ridgeogram that uses windows based on the physical location of the elements in the underlying sequence. Medians are calculated for a sequence of, in physical space regularly spaced, overlapping windows that now may contain a variable number of elements. P-values for the median are calculated using the presented exact formula based on the number of elements in the window. In Figure 1 and Additional file 4 results from calculations with the HTM [2] are shown that were obtained using the PosRidgeogram function of SigWinR. Because gene density is not equally distributed along the chromosome, as illustrated by the region near the centromere with low sample density in the lower Ridgeplot in Figure 1, the PosRidgeogram differs from the Ridgeogram. Because a position on the x-axis represents a position on the chromosome the PosRidgeogram can be interpreted in terms of position.

### Extending the algorithm

A consequence of the approach that takes physical position into account is that windows may contain an even number of elements. The SigWin-detector algorithm avoids even window sizes, because the previously presented exact formula [1] is only suited for windows with an uneven number of elements and the use of those uneven windows results in a Ridgeogram with sufficient resolution. To address this, we derived a formula for even windows that calculates the p-values associated with a certain median given sequence length and window size. This formula is presented in Additional file 5.

## Concluding Remarks

SigWinR is an R implementation of the grid-based SigWin-detector application for a desktop computer. It has an adequate performance and makes the SigWin algorithm available to a much wider audience than just the grid community. Also, with SigWinR, a number of improvements have been introduced, both in the algorithms used and the visualizations that can be produced. For future developments, we are considering parallelization of the SigWinR package.

## Availability and requirements

• **Project name**: SigWinR

• **Project home page**: 

• **Programming language**: R

• **Other requirements**: -

• **Download**: 

## Competing interests

The authors declare that they have no competing interests.

## Authors' contributions

WdL specified and implemented the SigWinR package.

HR, MAI and TB all worked on the specification of the SigWinR package and adapted it by discussing applicability of it with life scientists.

OB analyzed the Down syndrome gene expression data.

## Supplementary Material

Additional file 1 **RIDGES in HTM**. RIDGES in a human transcriptome map [2]. Click here for file

Additional file 2 **ROCAGEs on t-values**. ROCAGEs in Down Syndrome Brain expression data [4] for all chromosomes calculated by fold change of gene-expression log ratios of Down and control samples divided by their standard error (t-values). Click here for file

Additional file 3 **ROCAGEs on p-values**. ROCAGEs in Down Syndrome Brain expression data [4] for all chromosomes calculated by p-values on the Null Hypothesis of no differential expression between Down and control samples. Click here for file

Additional file 4 **Positional RIDGES in HTM**. Positional RIDGES (S2B) in a human transcriptome map [2]. Click here for file

Additional file 5 **An exact formula for the probability function**. An exact formula for calculating the probability given a median, window size and sequence length for even and uneven window sizes. Click here for file
