# Supplementary figures and images for: SigWinR; the SigWin-detector updated and ported to R
Source: BMC Res Notes. 2009 Oct 6;2:205. doi: 10.1186/1756-0500-2-205 (PMC2762987; doi:10.1186/1756-0500-2-205)

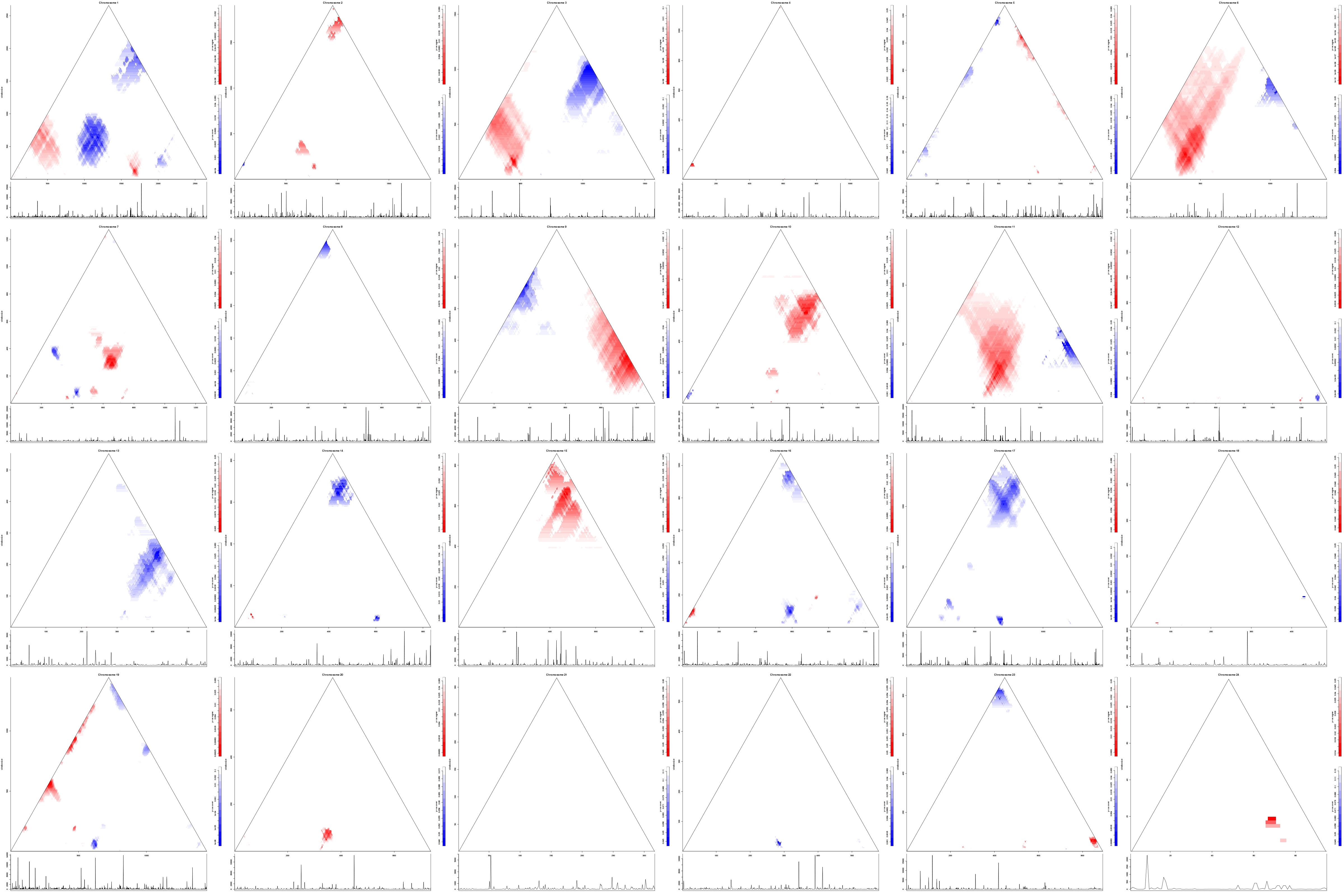

Supplement: Additional file 1 — RIDGES in HTM. RIDGES in a human transcriptome map [2]. [file 1756-0500-2-205-S1.PNG]

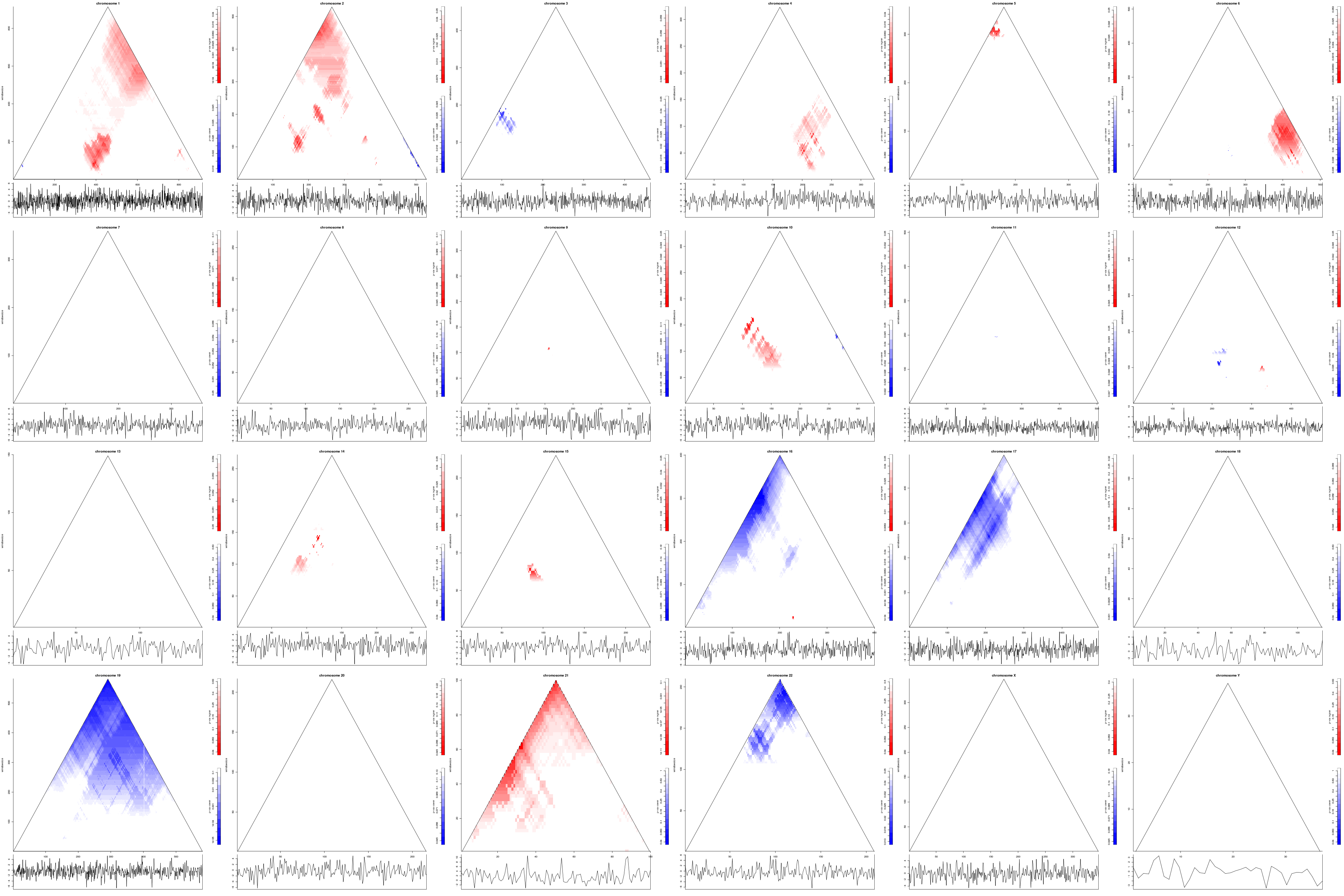

Supplement: Additional file 2 — ROCAGEs on t-values. ROCAGEs in Down Syndrome Brain expression data [4] for all chromosomes calculated by fold change of gene-expression log ratios of Down and control samples divided by their standard error (t-values). [file 1756-0500-2-205-S2.PNG]

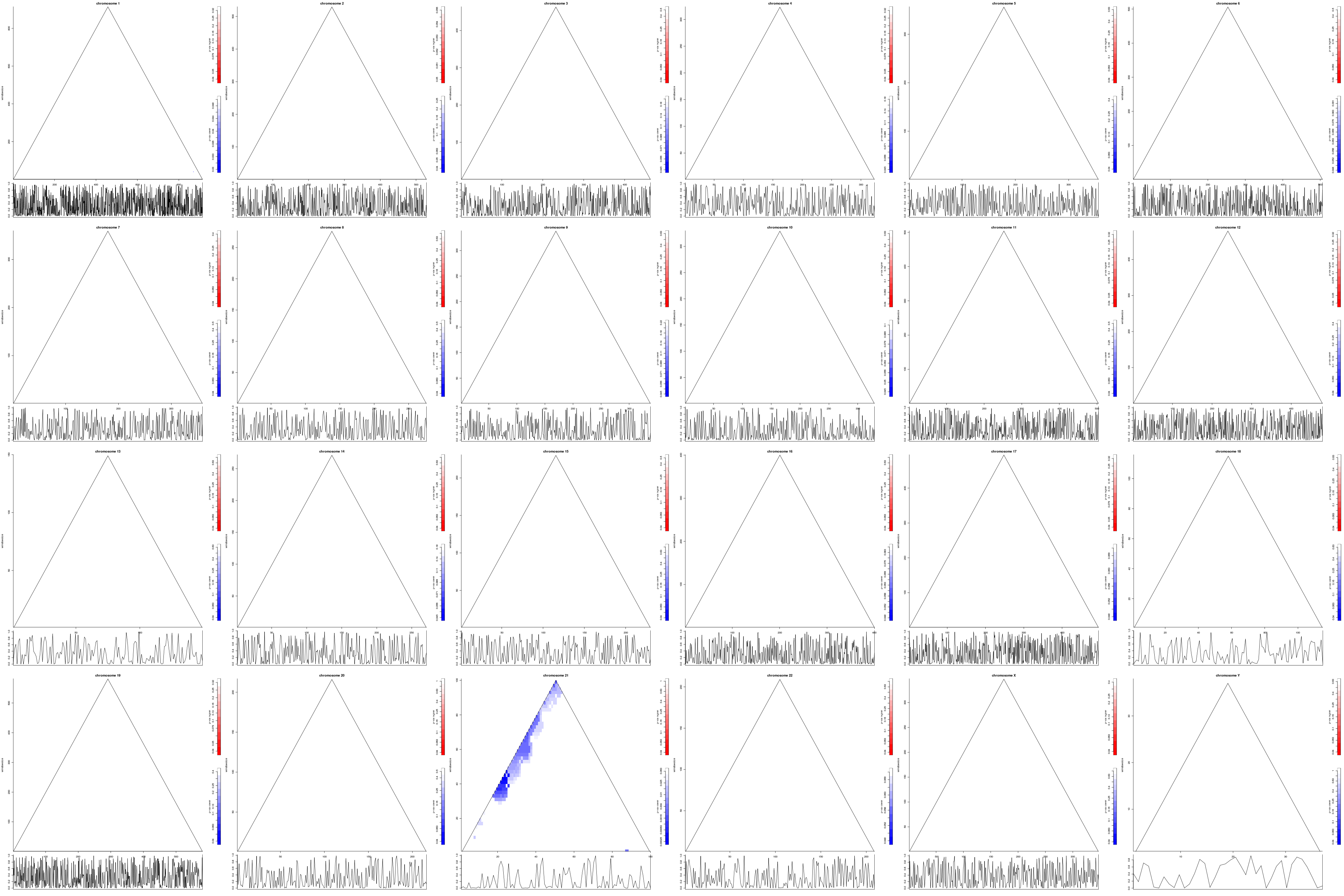

Supplement: Additional file 3 — ROCAGEs on p-values. ROCAGEs in Down Syndrome Brain expression data [4] for all chromosomes calculated by p-values on the Null Hypothesis of no differential expression between Down and control samples. [file 1756-0500-2-205-S3.PNG]

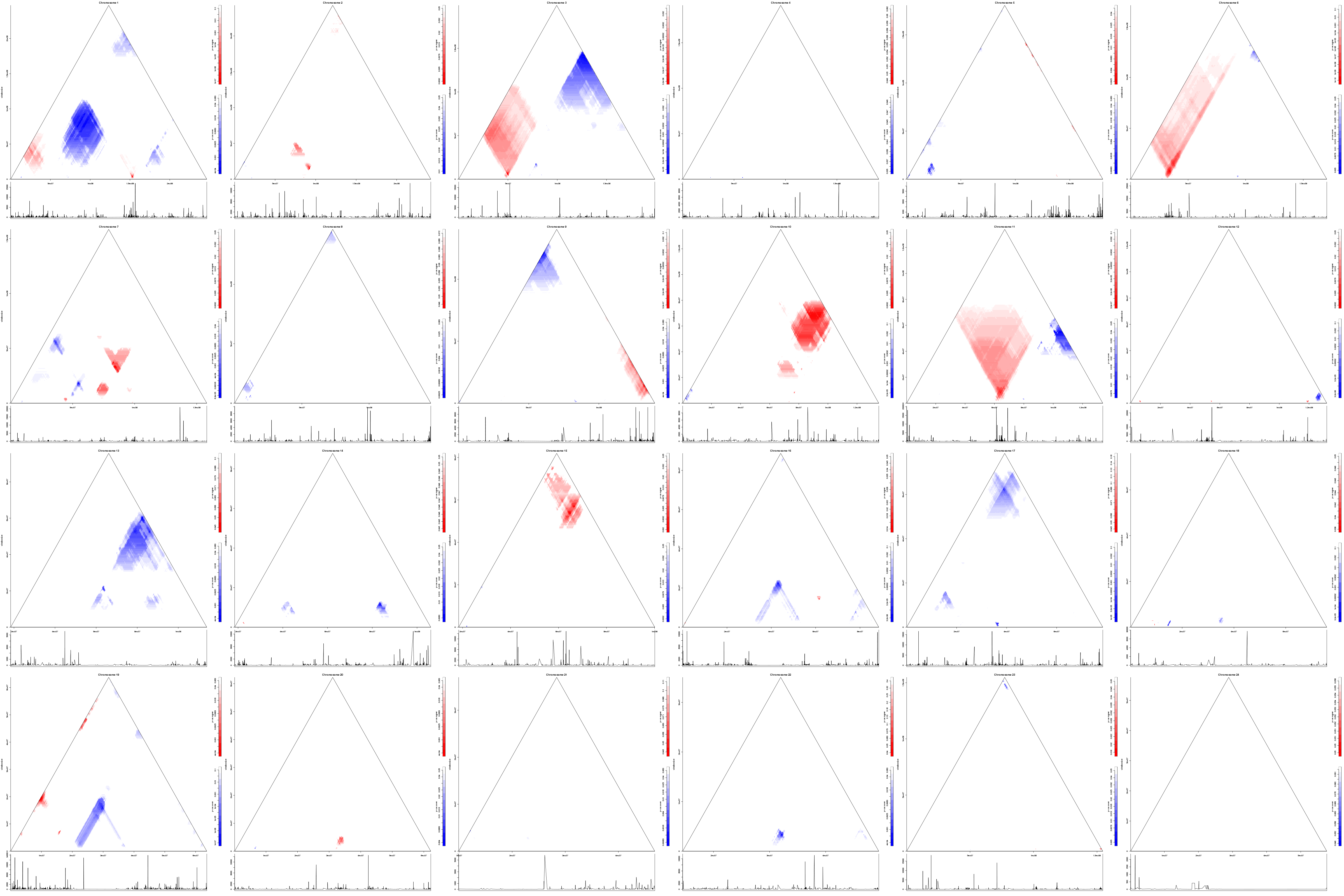

Supplement: Additional file 4 — Positional RIDGES in HTM. Positional RIDGES (S2B) in a human transcriptome map [2]. [file 1756-0500-2-205-S4.PNG]
