# Supplementary material for: SigWinR; the SigWin-detector updated and ported to R
Source: BMC Res Notes. 2009 Oct 6;2:205. doi: 10.1186/1756-0500-2-205 (PMC2762987; doi:10.1186/1756-0500-2-205)
Supplement: Additional file 5 — An exact formula for the probability function. An exact formula for calculating the probability given a median, window size and sequence length for even and uneven window sizes. [file 1756-0500-2-205-S5.PDF]

Additional file 1 To SigWinR; the SigWin-detector updated and ported to R

The formula describing the probability that a sample of size S from a sequence of ranks with length N has median r The derivation of the equation for odd window size can be found in Inda et al.

N	:	sequence length
S	:	window size
r	:	median of values in windows
$\hat{f}(r)$	:	probability sample of size S from N elements has median r

If S is odd:

$$\hat{f}(r) = \frac{\binom{r-1}{W}\binom{N-r}{W}}{\binom{N}{S}} \text{ where } W = \frac{s-1}{2}$$

If S is even the median is defined as the mean of the middle two elements of the ordered sequence.

$$\hat{f}(r) = \sum_{d=d_{Min}}^{d_{Max}} \frac{\binom{r-d-1}{W}\binom{N-d-r}{W}}{\binom{N}{S}} \text{ where } W = \frac{s-2}{2}$$

The values of d_{Min} and d_{Max} depend on the value of r:
If r is integer (the middle elements are both even or both odd):

$$\begin{aligned} d_{Min} &= 1 \\ d_{Max} &= \min(N-r-w, r-w-1) \end{aligned}$$

If r is non-integer (there is an even and an odd middle element):

$$\begin{aligned} d_{Min} &= 1/2 \\ d_{Max} &= \min(N-r+1/2-w, r+1/2-w-1) \end{aligned}$$
